# Supplementary material for: Identification of photoperiod-induced specific miRNAs in the adrenal glands of Sunite sheep (Ovis aries)
Source: Front Vet Sci. 2022 Jul 22;9:888207. doi: 10.3389/fvets.2022.888207 (PMC9354845; doi:10.3389/fvets.2022.888207)
Supplement: Supplementary file 1 [file Data_Sheet_1.ZIP › Supplementary file/miRDeep2 file.zip!/miRDeep2 file/Novel_Structure/SPLP42A3/Novel_387.pdf]

Provisional ID	:	NC_019460.2_7839
Score total	:	1.4
Score for star read(s)	:	-1.3
Score for read counts	:	0
Score for mfe	:	1.1
Score for randfold	:	1.6
Score for cons. seed	:	
Total read count	:	389
Mature read count	:	389
Loop read count	:	0
Star read count	:	0

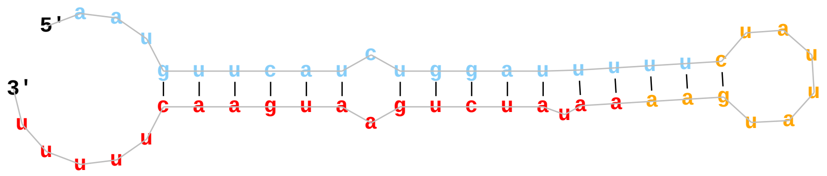

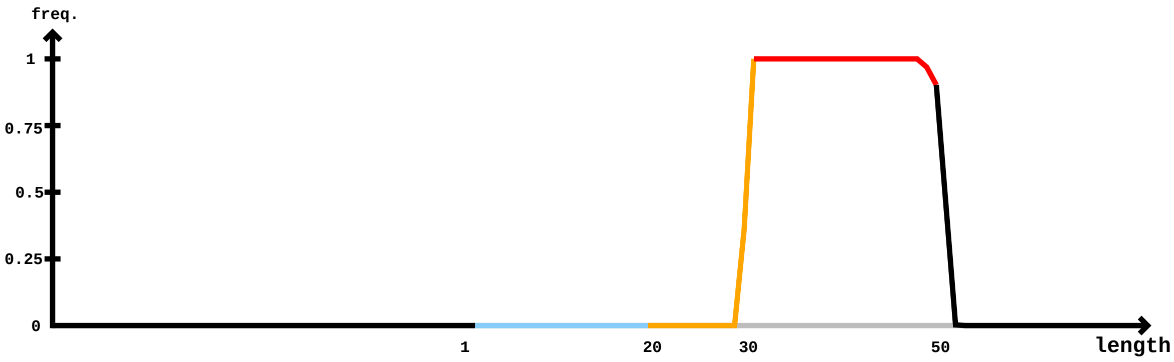

Star Mature

```
5'- aaagaaauauuuucaaaaauauuuuaauuuuaaguuggccaaaauguucaucuggauuuuucuauuaugaaaauaucugaaugaacuuuuuggccaaucaaauacagguuuc   -3'    exp
    ...(((((((((....)))))))))........(((((((((((.((((((.((((((((((......))))).))))).)))))).))))))))))).............     reads       mm          sample
    ......................................................................aaaAaucugaaugaacuuu.......................     12          1           seq
    ......................................................................aaaAaucugaaugaacuuuu......................     26          1           seq
    ......................................................................aaaAaucugaaugaacuuuuu.....................     78          1           seq
    ......................................................................aaauaucugaaugaacuuuuug....................     1           0           seq
    ......................................................................aaaAaucugaaugaacuuuuug....................     24          1           seq
    .......................................................................aaAaucugaaugaacuuuuu.....................     247         1           seq
    .......................................................................aauaucugaaugaacuuuuugg...................     1           0           seq
```
